# Identification of the Botanical Origin of Commercial Pine Nuts Responsible for Dysgeusia by Gas-Liquid Chromatography Analysis of Fatty Acid Profile

**DOI:** 10.1155/2011/316789

**Published:** 2011-03-10

**Authors:** Frédéric Destaillats, Cristina Cruz-Hernandez, Francesca Giuffrida, Fabiola Dionisi, Martine Mostin, Geert Verstegen

**Affiliations:** ^1^Nestlé Research Centre, Vers-chez-les-Blanc, 1000 Lausanne 26, Switzerland; ^2^Belgian Poison Control Centre, Hôpital Militaire Reine Astrid, Rue Bruyn, 1120 Bruxelles, Belgium

## Abstract

Over the last 10 years, complaints were increasingly reported from consumers that experienced dysgeusia following the consumption of pine nuts. In the present study, pine nuts samples (*N* = 16) from consumers that reported dysgeusia have been analyzed to identify the botanical origin of critical pine nuts samples. The fatty acid composition of the samples was performed, and diagnostic index values were used to identify the botanical origin of the samples. *Pinus armandii* nuts were identified in all the samples pure or in mixture with *P. koraiensis* nuts. *P. armandii* is not reported as edible pine nuts by the Food and Agriculture Organization (FAO). This study confirmed that consumption of *P. armandii* nuts may lead to dysgeusia. Based on the present study and previous work, we advise import companies to trade pine nuts from traditionally recognized species such as *P. pinea, P. sibirica, P. koraiensis,* or *P. gerardiana*.

## 1. Introduction

Pine nuts are traditionally used as ingredients in sauces such as pesto or in desserts. The main species consumed in Europe are *Pinus pinea*, *P. koraiensis, P. sibirica, *and *P. gerardiana*. Most of the commercial products are imported from Asian countries such as China, Korea, or Pakistan since local production is not sufficient and costly to answer the demand. Over the past 10 years, complaints have been reported by consumers who experienced dysgeusia and cacogeusia for several days following the consumption of imported pine nuts [1–4].

The chemical identity of the substance(s) responsible of the taste disturbances has not been found to date but a recent report from the French food safety agency [4] confirmed that no traces of residual contaminants known to be associated with bitter or metallic perception were found in these products. However, it is suspected that the botanical origin of the pine nuts found in the incriminated products might differ from traditionally consumed species [4]. We showed recently that the botanical origin of commercial pine nuts can be determined by analyzing the fatty acid composition since composition of several conifer seeds including *Pinus spp.* is known [5]. The special feature of *Pinus spp.* seed lipids is the occurrence of very specific fatty acids called Δ5-olefinic acids in different ratio and absolute content [6–8]. The method developed was used to analyze commercial products found in Europe, and results confirmed that pine nuts from special species not traditionally consumed such as *P. armandii* were found in mixture with *P. koraiensis* or as a sole botanical source [5].

However, it is not possible for the time being to formally associate the consumption of pine nuts from products containing specific* Pinus spp. *seeds to the occurrence of dysgeusia. In the present study, pine nuts samples collected by the Belgian Poison Center from consumers that reported taste disturbances have been analyzed. The primary objective of this study is to identify the botanical origin of critical pine nuts samples.

## 2. Materials and Methods

### 2.1. Samples and Reagents

Fifteen samples were obtained between July 2008 and April 2010 by the Belgian Poison Centre from consumers in Belgium, France, or the Netherlands who reported taste disturbance issues following the consumption of pine nuts. Consumers were asked to mail the pine nuts in their original container. All samples were from different distributors or had a different expiration dates. One sample was sent spontaneously by a consumer from New Zealand (Table 1). All solvents were HPLC grade, and hydrochloric acid in methanol (3N) was obtained from Supelco (Bellafonte, CA).

### 2.2. Sample Preparation

Pine nuts (about 1 g) were grounded in a mortar, and fatty acid methyl esters (FAME) were directly prepared without prior lipid extraction as previously described [5]. Briefly, grounded and dried nuts (100 mg) were mixed with methanol (2 mL), hydrochloric acid in methanol (3N, 2 mL), and hexane (2 mL) in tightly-closed test tubes. The methylation reaction was performed at 100°C for 1 h. After cooling-down to room temperature, water (2 mL) was added and sample vigorously vortexed for *ca.* 30 seconds. After centrifugation for 2 minutes at 3500 rpm, the hexane phase was diluted with an equal volume of fresh hexane, transferred in vials and analyzed by GLC.

### 2.3. Gas-Liquid Chromatography Analysis

Analysis of FAME was performed as described earlier [5] on a 7890 Agilent gas chromatograph (Agilent Technologies, Palo Alto, CA, USA), equipped with a fused-silica BPX-70 capillary column (10 m × 0.1 mm i.d., 0.2 *μ*m film thickness; SGE, Melbourne, Australia). Split injector (1800 : 1) and flame ionization detection (FID) systems were operated at 250°C and 300°C, respectively. Oven temperature programming was 50°C isothermal for 1 minute, increased to 180°C at 100°C/min, isothermal for 1 minute at this temperature, then increased to 220°C at 20°C/min and then to 250°C at 50°C/min. The carrier gas (H_2_) was maintained constant at 0.6 mL/min and the acquisition of the FID signal at 100 Hz.

## 3. Results

### 3.1. Description of the Samples and Reported Symptoms

The samples were obtained from consumers in Belgium, France, The Netherlands, and New Zealand. On four samples, the geographical origin of the pine nuts was mentioned on the container by the distributer: three of them came from China and one from “Siberia”. 

**Figure 1:**
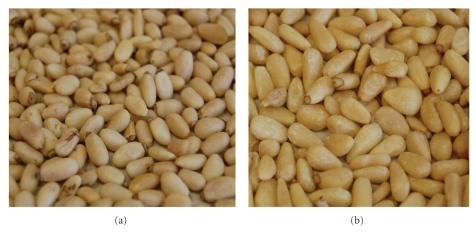
*Pinus armandii* (a) and *P. koraiensis *(b) pine nuts.

Samples 1, 4–8, 10, and 12–16 had a similar appearance with length of 8–11 mm and a width of 4–6 mm. They are rounded and have grayish-beige color (see Figure 1(a) for a representative picture). Careful visual examination of the samples 2, 3, 9, and 11 revealed the occurrence of two populations of seeds, one similar to the previous samples and a second population of pine nuts that were larger with a length of 10–13 mm and width of 6–8 mm. The color was brighter, more yellowish without grey tones (see Figure 1(b) for a representative picture).

### 3.2. Analysis of the Fatty Acid Profile

The morphology of the pine nuts in samples 1, 4–8, 10, and 12–16 was similar while careful visual examination of the samples 2, 3, 9, and 11 revealed the occurrence of two populations of seeds. These populations were characterized by different diameter and length and therefore two subsets were prepared and treated separately. The fatty acid profile of samples containing *a priori* only one type of seeds is reported in Table 2. Results obtained from products containing two types of pine nuts are reported in Table 3. The pine nuts subtype annotated in Table 3 as subtype A is similar to pine nuts from samples 1, 4–8, 10, and 12–16 but rounder and shorter than the subtype B. Fatty acid profile of the samples was determined according to previous publication by fast GLC analysis of FAME prepared by acid catalysis directly from pulverized pine nut samples [5]. The results were expressed as relative % (g per 100 g of total fatty acid), and botanical origin was determined using the formula of the diagnostic index (DI) as previously described:

(1) $$\begin{array}{c} \text{DI}=\left[\frac{\left(5,9\text{–}18:2+5,9,12\text{–}18:3+5,11,14\text{–}20:3\right)}{\left(18:1\;\text{n-}9\;\text{and}\;\text{n-}7+18:2\;\text{n-}6+20:2\;\text{n-}6\right)}\right]\times 10. \end{array}$$

Typical DI values calculated from literature data or from authentic samples have been reported previously [5]. The identification of the botanical origin of pine nuts using this DI is based on the fact that pine nuts contain specific polyunsaturated fatty acids characterized known as Δ5-olefinic acids. The occurrence and level of these fatty acids have been successfully used as taxonomic factors to discriminate conifer within and between different genders [6–8]. 

The identification conducted in the present study from the results provided in Tables 2 and 3 is provided in Table 1. DI values for samples 1, 7, 10, and 13–16 ranged from 2.79 to 2.99 (mean value 2.91, standard deviation 0.08) and are very comparable to the reference value for *P. armandii* (2.92). Similar fatty acid composition and DI were obtained for the subtype A of pine nut samples 2, 3, 9, and 11 that had DI values ranging from 3.02 to 2.85 (mean value 2.96, standard deviation 0.08). These 11 samples were all identified to contain *P. armandii* seeds as the sole source of pine nuts or in mixture. Analyses of the pine nut subtype B in samples 2, 3, 9, and 11 based on the DI value that ranged from 2.37 to 2.61 (mean value 2.47, standard deviation 0.10) lead to the confirmation that theses pine nuts have been obtained from *P. koraiensis* (reference value 2.38). This species is traditionally used as ingredient for the preparation of sauces and desert or used roasted or crude in salads [9].

Samples 4–6, 8, and 12 have DI values (mean value 3.16, standard deviation 0.08) in average higher than samples 1, 7, 10, and 13–16 (mean value 2.91, standard deviation 0.08). DI values of most common *Pinus spp.* possibly exported from Asia were reported in a previous study. The reference values calculated from literature data [6] in the range observed for samples 4–6, 8, and 12 are *P. sibirica* (3.03) and *P. massoniana* (3.55). Therefore, it can then be hypothesized that samples 4–6, 8, and 12 differ from samples 1, 7, 10, and 13–16 identified as containing *P. armandii* nuts. However, the level of sciadonic acid in these samples (mean value 1.63, standard deviation 0.20) differs from typical values reported in the literature for *P. sibirica* (<1%) or in *P. massoniana* (>3%) [6]. Moreover, *P. sibirica* is characterized by lower level of linoleic and sciadonic acids and higher level of pinolenic (5,9,12–18 : 3) acid compared to *P. armandii* [6]. In addition, *P. massoniana* is characterized by lower level of octadecenoic (18 : 1 n-9 + n-7) acids and higher levels of linoleic, pinolenic, and sciadonic acids and the seeds are smaller (<4 mm) than those found in the different samples [10]. The overall fatty acid composition of samples 4–6, 8, and 12 is close to those of samples 1, 7, 10, and 13–16 (Table 2) but slight variations leading to a variation of the DI values for samples 4–6, 8, and 12, in average + 9% compared to samples 1, 7, 10, and 13–16, are observed. These differences are explained by a lower content of the sum of octadecenoic, linoleic, and eicosadienoic acids (in average −1% compared to samples 1, 7, 10, and 13–16) and a higher value for the sum of the three main Δ5-olefinic acids (in average +7% compared to samples 1, 7, 10, 13–16). These variations are low and might be due to the natural variation of the different fatty acids considered in the calculation of the DI value. Therefore, it can be concluded that samples 4–6, 8, and 12 which slightly differ from samples 1, 7, 10, and 13–16 are as well nuts from *P. armandii* (Table 1).

## 4. Discussion

The main symptom that was reported for these samples was dysgeusia, typically starting 1 or 2 days after consumption and lasting 5–10 days. The quantity eaten was usually small (less than 10 grams). The victims described a permanent, mainly bitter taste, accentuated by consumption of bread, coffee, tea, or wine. In one occasion, slight abdominal discomfort was reported. We are not aware of any other systemic symptoms. The correlation between the taste disturbances and pine nuts consumption was usually not made by the victims. They contacted the Poison centre looking for a cause for their taste disturbances. 

All the samples reported by consumers who suffered from dysgeusia have shown to contain nuts from *Pinus spp*. identified as *P. armandii* in mixture or not with *P. koraiensis* nuts. These findings suggest that the dysgeusia following the consumption of pine nuts is caused by pine species contamination, more specifically by *P. armandii*. The mixtures with *P. koraiensis* is probably caused by the common geographic origin of both species. *P. armandii* is used for building purposes in greater China and not to harvest seeds as a first instance. This element is very important to consider in order to identify the causality between the consumption of these nuts and the symptoms reported by the consumers. Indeed, one can speculate that genuine intrinsic constituents of *P. armandii* nuts can be responsible of the dysgeusia symptoms in humans. However, the occurrence of chemical agents or process conditions used for the isolation of the raw seeds or the shell removal might also be responsible for the occurrence or formation of the undesired molecule(s) leading to dysgeusia symptoms. Process conditions might lead to reactions such as O-acyl hydrolysis, oxidative, or thermo-oxidative degradation and generation of products that can cross-react with processing agent or contaminants. Therefore, several hypotheses can be generated to unravel this pending concern with imported pine nuts. A critical analysis of the putative causes has been recently published on line on the Institute of Food Technologists [11].

In depth characterization of the chemical composition of *P. armandii* nuts will be necessary to identify the molecule(s) responsible of dysgeusia. However, we advice companies to redirect their pine nuts supply towards *Pinus spp*. traditionally used and recognized by FAO such as *P. pinea*, *P. koraiensis, P. sibirica, *and *P. gerardiana *[9]. Additionally we think that the European food regulatory authorities should introduce a positive list of edible pine nuts in the legislation.

## Figures and Tables

**Table 1:** Information and identification of the botanical origins of 16 different commercial products collected from subjects who experienced dysgeusia. Determination of the botanical origin was performed by analysis of the fatty acid profile according to published method [5].

Sample code	Collection period	Country of the trademark	Symptoms	Botanical identity determined from fatty acid profile
1	July 2009	Belgium	dysgeusia, abdominal dyscomfort	*Pinus armandii*
2	October 2010	France	dysgeusia	Mixture of *Pinus koraiensis* and *Pinus armandii *
3	December 2008	Netherlands	dysgeusia	Mixture of *Pinus koraiensis* and *Pinus armandii *
4	—	Netherlands	dysgeusia	*Pinus armandii*
5	—	Netherlands	dysgeusia	*Pinus armandii*
6	July 2010	Netherlands	dysgeusia	*Pinus armandii*
7	July 2008	Netherlands	dysgeusia	*Pinus armandii*
8	July 2008	Netherlands	dysgeusia	*Pinus armandii*
9	July 2008	Netherlands	dysgeusia	Mixture of *Pinus koraiensis* and *Pinus armandii *
10	July 2008	—	dysgeusia	*Pinus armandii*
11	March 2010	Belgium	dysgeusia	Mixture of *Pinus koraiensis* and *Pinus armandii *
12	July 2010	Netherlands	dysgeusia	*Pinus armandii*
13	April 2010	New-Zealand	dysgeusia	*Pinus armandii*
14	April 2010	Belgium	dysgeusia	*Pinus armandii*
15	April 2010	Belgium	dysgeusia	*Pinus armandii*
16	April 2010	Belgium	dysgeusia	*Pinus armandii*

**Table 2:** Fatty acid profile of pine nuts samples containing only one type of pine nuts. Diagnostic index (DI) value: DI = [(5, 9–18 : 2 + 5, 9, 12–18 : 3 + 5,11,14–20 : 3) / (18 : 1 n-9 and n-7 + 18 : 2 n-6 + 20 : 2 n-6)] × 10. Results of duplicate analysis expressed as g per 100 g of fatty acids.

Sample code	1		4		5		6		7		8		10		12		13		14		15		16	
Fatty acid	MV	SD	MV	SD	MV	SD	MV	SD	MV	SD	MV	SD	MV	SD	MV	SD	MV	SD	MV	SD	MV	SD	MV	SD
16:0	4.95	0.09	4.58	0.09	4.57	0.13	4.72	0.13	5.08	0.19	4.79	0.12	4.90	0.17	4.81	0.02	5.00	0.10	4.54	0.04	4.65	0.06	4.93	0.02
16:1 n-9	0.05	0.00	0.05	0.00	0.05	0.00	0.03	0.02	0.03	0.01	0.05	0.00	0.04	0.01	0.05	0.01	0.03	0.00	0.06	0.00	0.06	0.00	0.06	0.00
16:1 n-7	0.07	0.00	0.07	0.01	0.09	0.02	0.05	0.03	0.06	0.01	0.07	0.02	0.06	0.01	0.09	0.01	0.08	0.00	0.08	0.00	0.10	0.01	0.12	0.01
17:0	0.05	0.01	0.07	0.01	0.06	0.00	0.06	0.01	0.06	0.02	0.06	0.02	0.08	0.02	0.04	0.00	0.05	0.01	0.04	0.00	0.05	0.01	0.04	0.00
17:1 n-9	0.04	0.01	0.04	0.01	0.04	0.01	0.04	0.01	0.03	0.00	0.03	0.02	0.03	0.01	0.03	0.01	0.03	0.01	0.03	0.00	0.04	0.00	0.04	0.01
18:0	1.82	0.01	2.04	0.07	1.84	0.23	1.70	0.07	2.58	0.00	1.66	0.15	2.43	0.24	1.78	0.01	2.13	0.25	2.09	0.02	1.98	0.02	1.99	0.01
18:1 n-9 + n-7	20.88	0.51	22.79	0.23	22.64	1.19	19.81	1.24	24.55	0.40	20.14	0.98	24.37	0.35	22.65	0.17	25.31	0.24	23.85	0.28	23.31	0.42	24.60	0.13
5,9–18:2	2.13	0.13	3.81	0.28	3.65	0.33	2.42	0.11	2.08	0.15	2.38	0.02	2.37	0.28	3.04	0.02	2.38	0.09	3.68	0.00	3.69	0.02	3.94	0.02
18:2 n-6	48.35	0.21	46.23	0.21	46.10	0.71	48.12	0.56	44.69	0.25	48.19	0.76	44.47	0.69	46.29	0.04	45.03	0.57	45.89	0.29	46.25	0.32	45.23	0.04
5,9,12–18:3	17.11	0.28	16.39	0.16	16.31	0.85	18.39	0.41	16.42	0.60	17.71	0.77	17.09	0.42	17.02	0.11	16.26	0.25	15.77	0.03	15.81	0.17	14.88	0.14
18:3 n-3	0.26	0.00	0.22	0.01	0.22	0.02	0.28	0.04	0.22	0.02	0.31	0.05	0.23	0.01	0.23	0.01	0.21	0.03	0.24	0.00	0.27	0.01	0.33	0.00
20:0	0.36	0.01	0.36	0.01	0.35	0.04	0.31	0.01	0.41	0.02	0.32	0.03	0.40	0.02	0.32	0.01	0.33	0.05	0.41	0.01	0.41	0.00	0.38	0.00
20:1 n-9	1.06	0.04	0.93	0.06	1.12	0.08	1.06	0.07	1.31	0.02	1.07	0.07	1.15	0.02	1.01	0.02	1.04	0.08	0.93	0.00	0.94	0.02	0.97	0.02
5,11–20:2	0.16	0.01	0.19	0.01	0.22	0.02	0.16	0.01	0.16	0.00	0.18	0.02	0.15	0.02	0.17	0.00	0.13	0.02	0.17	0.01	0.19	0.01	0.19	0.01
20:2 n-6	0.67	0.00	0.55	0.03	0.68	0.04	0.73	0.10	0.69	0.00	0.71	0.03	0.61	0.02	0.60	0.03	0.55	0.03	0.55	0.01	0.55	0.01	0.54	0.00
5,11,14–20:3	1.66	0.04	1.36	0.01	1.68	0.09	1.71	0.20	1.21	0.00	1.88	0.19	1.22	0.07	1.50	0.04	1.11	0.04	1.24	0.01	1.28	0.02	1.32	0.02
7,11,14–20:3	0.12	0.02	0.10	0.01	0.14	0.02	0.16	0.03	0.13	0.01	0.15	0.02	0.12	0.00	0.13	0.01	0.09	0.01	0.10	0.01	0.10	0.00	0.10	0.01
22:0	0.13	0.00	0.11	0.00	0.14	0.01	0.12	0.01	0.13	0.01	0.13	0.01	0.13	0.00	0.11	0.00	0.09	0.02	0.15	0.00	0.15	0.00	0.15	0.00
Other FA	0.13	0.01	0.15	0.01	0.11	0.01	0.10	0.01	0.13	0.02	0.12	0.01	0.07	0.03	0.11	0.03	0.11	0.02	0.18	0.00	0.18	0.01	0.17	0.00

DI	2.99	0.08	3.10	0.06	3.12	0.13	3.28	0.10	2.82	0.07	3.18	0.11	2.98	0.12	3.10	0.03	2.79	0.03	2.94	0.00	2.96	0.02	2.86	0.02

**Table 3:** Fatty acid profile of pine nuts obtained from samples containing two types of pine nuts named A and B. Diagnostic index (DI) value: DI = [(5, 9–18 : 2 + 5, 9, 12–18 : 3 + 5, 11, 14–20 : 3) / (18 : 1 n-9 & n-7 + 18 : 2 n-6 + 20 : 2 n-6)] × 10. Results of duplicate analysis expressed as g per 100 g of fatty acids.

Sample code	2				3				9				11			
Seed type	A		B		A		B		A		B		A		B	
Fatty acid	MV	SD	MV	SD	MV	SD	MV	SD	MV	SD	MV	SD	MV	SD	MV	SD
16:0	4.69	0.03	5.29	0.24	4.60	0.05	5.17	0.03	4.61	0.07	5.18	0.20	4.29	0.02	4.98	0.13
16:1 n-9	0.06	0.00	0.02	0.01	0.03	0.00	0.01	0.00	0.03	0.00	0.02	0.00	0.04	0.01	0.02	0.00
16:1 n-7	0.11	0.01	0.08	0.00	0.05	0.00	0.06	0.01	0.07	0.01	0.06	0.03	0.08	0.01	0.07	0.02
17:0	0.08	0.02	0.06	0.01	0.06	0.00	0.07	0.01	0.08	0.01	0.05	0.01	0.09	0.01	0.07	0.01
17:1 n-9	0.04	0.00	0.03	0.00	0.03	0.00	0.03	0.00	0.03	0.00	0.03	0.00	0.05	0.02	0.03	0.00
18:0	1.94	0.01	2.30	0.21	2.39	0.02	2.17	0.03	2.35	0.03	2.09	0.05	2.06	0.01	2.34	0.04
18:1 n-9 + n-7	23.12	0.18	26.51	0.86	23.22	0.20	26.81	0.59	24.24	0.08	26.52	0.08	24.07	0.42	25.89	0.36
5,9-18:2	3.63	0.06	2.21	0.12	2.01	0.03	2.12	0.02	2.48	0.02	2.22	0.08	2.50	0.02	2.32	0.01
18:2 n-6	46.26	0.11	45.62	0.27	45.90	0.04	45.19	0.15	45.51	0.04	45.15	0.25	45.53	0.49	45.68	0.21
5,9,12–18:3	15.99	0.10	14.06	0.54	17.93	0.26	14.57	0.40	16.17	0.17	14.49	0.45	16.64	0.17	14.73	0.05
18:3 n-3	0.24	0.00	0.17	0.00	0.22	0.01	0.18	0.01	0.21	0.01	0.19	0.01	0.17	0.00	0.14	0.01
20:0	0.35	0.01	0.37	0.01	0.36	0.00	0.37	0.01	0.38	0.02	0.38	0.01	0.37	0.00	0.38	0.01
20:1 n-9	0.93	0.01	1.22	0.05	1.06	0.01	1.19	0.02	1.22	0.01	1.32	0.01	1.23	0.04	1.30	0.00
5,11–20:2	0.19	0.00	0.12	0.01	0.13	0.00	0.12	0.01	0.17	0.00	0.14	0.01	0.20	0.00	0.11	0.01
20:2 n-6	0.53	0.02	0.63	0.04	0.56	0.01	0.61	0.01	0.68	0.02	0.66	0.00	0.68	0.02	0.67	0.00
5,11,14–20:3	1.45	0.04	1.01	0.02	1.08	0.00	1.02	0.01	1.39	0.00	1.17	0.00	1.59	0.03	0.98	0.01
7,11,14–20:3	0.11	0.00	0.08	0.00	0.12	0.00	0.07	0.00	0.13	0.00	0.09	0.00	0.16	0.00	0.08	0.00
22:0	0.12	0.00	0.11	0.00	0.11	0.01	0.11	0.01	0.11	0.01	0.14	0.02	0.12	0.00	0.12	0.01
Other FA	0.15	0.01	0.11	0.01	0.13	0.02	0.10	0.01	0.14	0.03	0.10	0.03	0.14	0.20	0.10	0.05

DI	3.01	0.04	2.37	0.11	3.02	0.04	2.44	0.07	2.85	0.02	2.47	0.08	2.95	0.03	2.50	0.00
